# Direct oral anticoagulants and its implications in dentistry. A review of literature

**DOI:** 10.4317/jced.54004

**Published:** 2017-11-01

**Authors:** Neus Lanau, Javier Mareque, Lluis Giner, Michel Zabalza

**Affiliations:** 1 DDS, PhD Student. Faculty of Dentistry. Universitat Internacional de Catalunya, Spain; 2 MD, DDS, PhD. Vice-dean for Research. Faculty of Dentistry. Universitat Internacional de Catalunya, Spain; 3 MD, DDS, PhD. Dean of the Faculty of Dentistry. Universitat Internacional de Catalunya, Spain; 4 MD, PhD. Faculty of Medicine and Dentistry. Universitat Internacional de Catalunya, Spain

## Abstract

**Background:**

Four novel direct oral anticoagulants (DOACs) named dabigatran, rivaroxaban, edoxaban and apixaban have been recently introduced to overcome some of the drawbacks of existing anticoagulants. They have less interactions and do not require routine monitoring. However, there is not enough scientific data about the protocol to apply in these patients on DOACs undergoing dental treatment. Thus is necessary to evaluate the potential bleeding risk of these drugs, the possibility of thromboembolic events occurring if they are withdrawn or the need to change to heparin previously.

**Material and Methods:**

A comprehensive search of the PubMed, Scopus and ISI Web of Science databases was conducted to identify studies that evaluated the relationship between direct oral anticoagulants and dental procedures. The quality of the reported information was assessed following the PRISMA statement.

**Results:**

Eleven studies that met the inclusion criteria were included in the review: 2 randomized clinical trials, 3 prospective studies, 3 retrospective studies, 2 case series and 1 case report.

**Conclusions:**

DOACs are safe drugs in terms of bleeding. The possible postoperative bleeding complications are manageable with conventional haemostasis measurements. The bridging approach with heparin does not seem to be recommended. Consensus among the professionals involved in the management of the patient is fundamental in invasive dental treatments and in complex patients.

** Key words:**Oral anticoagulants, DOAC, NOAC, dabigatran, rivaroxaban, apixaban, edoxaban, bleeding, oral surgery.

## Introduction

Nowadays, anticoagulation therapy is required by a lot of patients to prevent, treat or reduce the risk of thromboembolism in atrial fibrillation, treatment of venous thromboembolism, cerebro-vascular accidents, ischaemic heart disease, myocardial infarction, pulmonary embolism and in prevention of thromboembolism after hip and knee replacement or stent placement, bypass surgery and prosthetic heart valve placement (1-3).

Historically, vitamin K antagonists such as warfarin and acenocumarol, have been the oral anticoagulants of choice (4). However they have some disadvantages such as low therapeutic index, delayed onset of action, many drug and food interactions and difficult pharmacological management since they require a regular monitoring and adjustment (5,6).

In the recent years, Direct Oral Anticoagulants (DOACs) have been introduced in order to eliminate some of these disadvantages. The first four DOACs are: dabigatran, rivaroxaban, apixaban and edoxaban ( Table 1). This novel agents target specific proteins or proteases of the coagulation cascade such as thrombin or activated factor Xa (2,5). They have an immediate onset of action, more predictable pharmacokinetics, less drug interactions than warfarin and a short half-life (7). The most important disadvantage is that there is no specific agent to reverse the anticoagulant effect of DOACs.

**Table 1:**
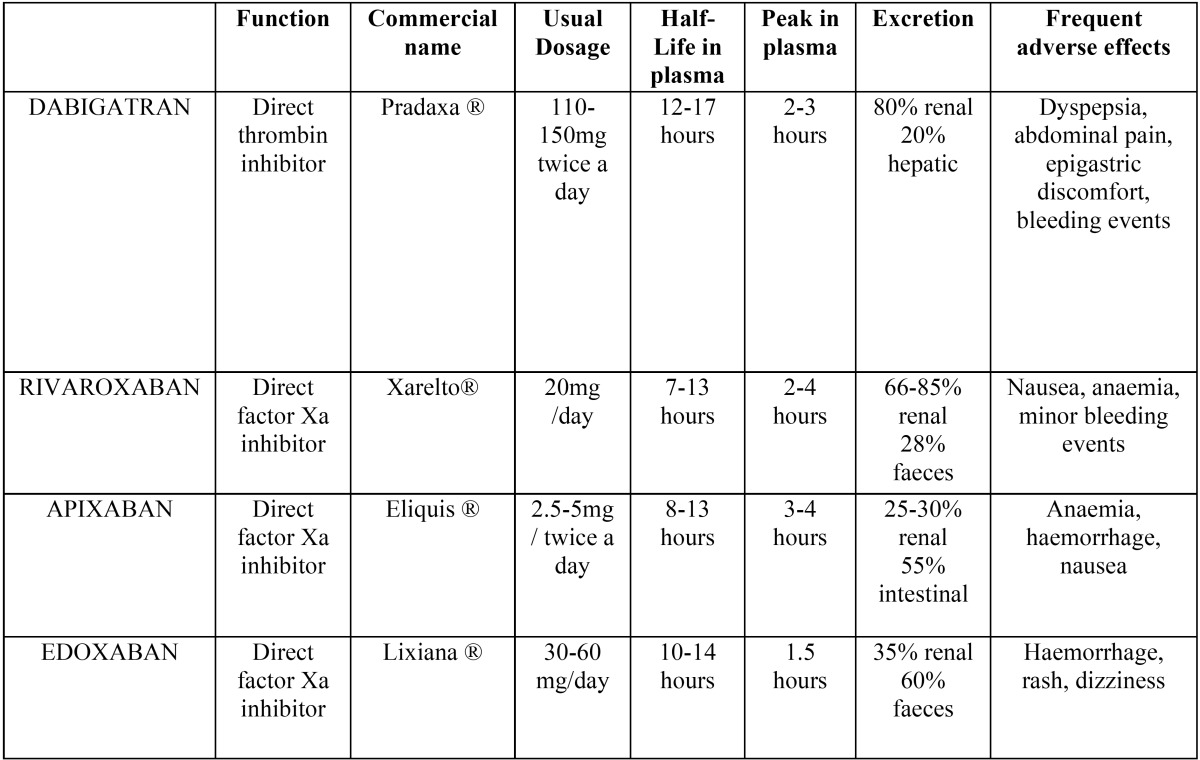
Pharmacological properties of DOACs.

-Dabigatran

Dabigatran etexilate is a low molecular weight prodrug of dabigatran, a molecule that inhibits free and clot-bound thrombin (5). It has short half-life (12-17 hours) and rapid onset of action, its peak in plasma takes place 2-3 hours after administration, which is one or twice per day (4,5,8,9). 80% is excreted renally and the remaining 20% is cleared by hepatic metabolism. So in patients with reduced renal function, the dose needs to be adjusted depending on the creatinine clearance.

One of the major advantages is that does not require routine monitoring of anticoagulant effect, due to its predictable pharmaco-kinetics and dose response. However, in some situations it may be necessary an assessment of anticoagulation. Prothrombin time (PT) expressed as the INR, which most dentists are familiar with, is not a sensitive test and is not normally affected by dabigatran. An accurate test that can be useful is thrombin clotting time (TT) (3-5).

The most frequent adverse effects experienced by the patients (>15% of patients) are gastritis type symptoms such as dyspepsia, abdominal pain and epigastric discomfort. Minor bleeding events were reported by 8-33% of the patients and major bleeding events by ≤6% of the patients (3,10,11).

The major disadvantage is that there is no specific antidote or reversal agent for an emergency situation. Dabigatran can be partially reversed with haemodialysis (5).

-Rivaroxaban

Rivaroxaban is an orally administered, selective, reversible, direct inhibitor of activated factor X (factor Xa) anticoagulant (3,4). It inhibits directly factor Xa, therefore it interrupts the extrinsic and intrinsic coagulation pathways. The plasma concentration peak of rivaroxaban is 2-4 hours after administration and its half-life in plasma is 7-13 hours. It is excreted basically in the urine (67-85%). It may be necessary some dose adjustments in patients with severe renal impairment if the plasma levels increase (8,12).

Like dabigatran, it is not required a routine monitoring. However, in an emergency situation an anti-factor Xa assay can be useful (3,4).

The adverse effects are experienced by 1-10% of the patients. Major bleeding has been reported in 1-2% of the patients, and minor bleeding in 4-7% (4,13).

The most important disadvantage is that there is no specific agent to reverse the anticoagulant effect of rivaroxaban. For a minor haemorrhage, due to its short half-life, discontinuation of the treatment should be enough. However, in major haemorrhages it may be necessary a blood transfusion (4,8).

-Apixaban

Apixaban is a direct inhibitor of activated factor X (it blocks the active site of Xa), like rivaroxaban consequently it has very similar properties (12).

Apixaban has a rapid onset of action: the plasma concentration peak is 3-4 hours after administration and its half-life in plasma is 8-13 hours. 25-30% is excreted by renal metabolism (12).

The most frequent adverse effects are anaemia, haemorrhage and nausea. Like rivaroxaban it is not required a routine monitoring. Anyhow, in an emergency situation, a calibrated anti-Xa assay is the most sensitive test and in major haemorrhages it may be necessary the administration of prothrombin complex concentrates (12).

-Edoxaban

Edoxaxaban, such as Apixaban and Rivaroxaban, is also a direct inhibitor of activated factor X (12). The plasma concentration peak of edoxaban is 1.5 hours after administration and its half-life in plasma it is 10-14 hours. It is 35% excreted renally and 60% in faeces (12).

The most frequent adverse effects are haemorrhage, rash and dizziness. As rivaroxaban and apixaban this drug does not require a routine monitoring. However, in an emergency situation a calibrated anti-Xa assay may be useful and in case of severe haemorrhages, it may be necessary the administration of prothrombin complex concentrates (12).

These new anticoagulants have been progressively prescribed in the treatments of patients who go to the dental office without clear indications, warnings, recommendations or protocols that allow the dentists to perform their procedures with the greatest professionalism. Dentists are familiarized with traditional anticoagulants (vitamin K inhibitors) and the methods of monitoring tests such as International normalized ratio (INR) and thus determine the safety, whether to perform or not an invasive dental treatment that involves bleeding (13-31). However, there are not enough reliable clinical trials and consensus about the protocol to apply in patients on DOACs undergoing dental treatment. Issues such as previous discontinuation of the DOAC, the possibility of bridging with heparin, prothrombotic risk, and possibilities of bleeding appear more frequently among dentistry professionals.

Therefore, it is necessary to address all these issues and to summarize them through a review of the literature available for the optimal management of these patients in the dental office.

## Material and Methods

-Study selection

A comprehensive search of the PubMed, Scopus databases and ISI Web of Science databases from its inception through October 2016 was conducted to identify studies that evaluated the relationship between direct oral anticoagulants and dental procedures. We queried MeSH terms and the article text for the following search terms: (‘dentistry’) OR (‘oral surgery’) AND (‘DOAC’, ‘NOAC’, ‘TSOAC’, ‘dabigatran’, ‘apixaban’, ‘rivaroxaban’, ‘edoxaban’). The selection criteria were all kind of articles (rando-mized clinical trials, cohort studies, case-control studies, case series and case reports), performed in adult patients under treatment with any kind of DOAC drug who underwent a dental procedure that implied any kind of bleeding. The search strategy was limited to articles published in English and human studies.

The articles returned by this search were manually screened, first on the basis of the title, then the abstract and finally the complete manuscript, to assess their appropriateness for inclusion in the literature review. References cited in these articles were also reviewed to identify additional published articles not identified by the database search.

-Data extraction

Two investigators (MZ and NL) independently extracted data. The data extracted included information about the study design characteristics, the outcomes assessed, the cohort characteristics, and the reported results. Disagreements were resolved through consensus.

The quality of the reported information included in each article was assessed following the PRISMA statement (32) for the improvement of the publication of reviews.

## Results

**Figure 1:**
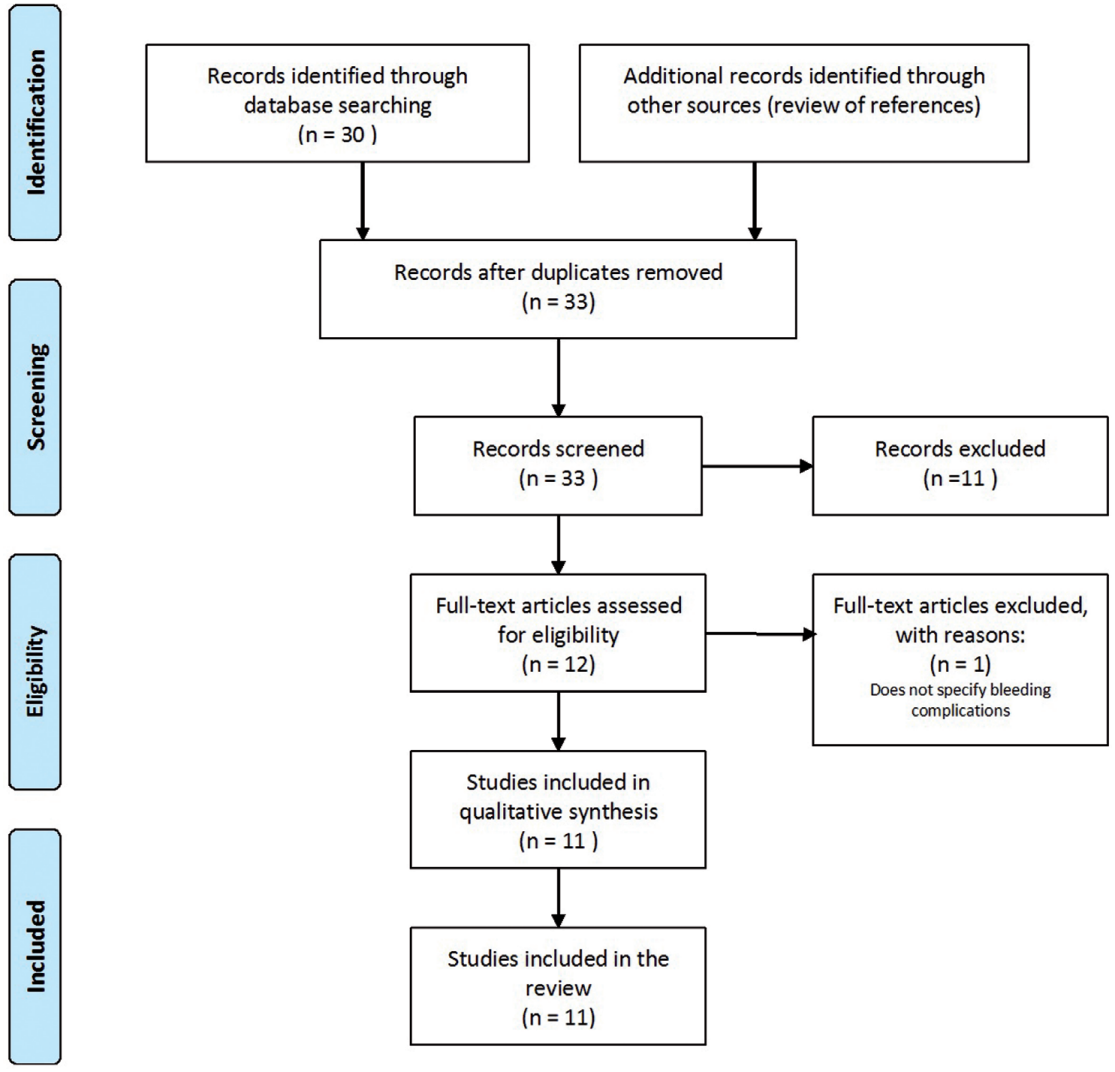
Diagram flow of the selection process of the studies included.

The process of selection of studies for inclusion in the review is summarised in figure 1 (diagram flow). The database search identified 30 unique citations, of which 28 were judged to be of potential interest on the basis of the title. On the basis of the abstract, 12 studies were reviewed in their entirety. One study was excluded because it did not specify the bleeding complications after dental treatments. Five additional studies were identified after review of the references.

Finally, 11 studies that met the criteria described above were included in the review (see Tables  Table 2- Table 4).

**Table 2:**
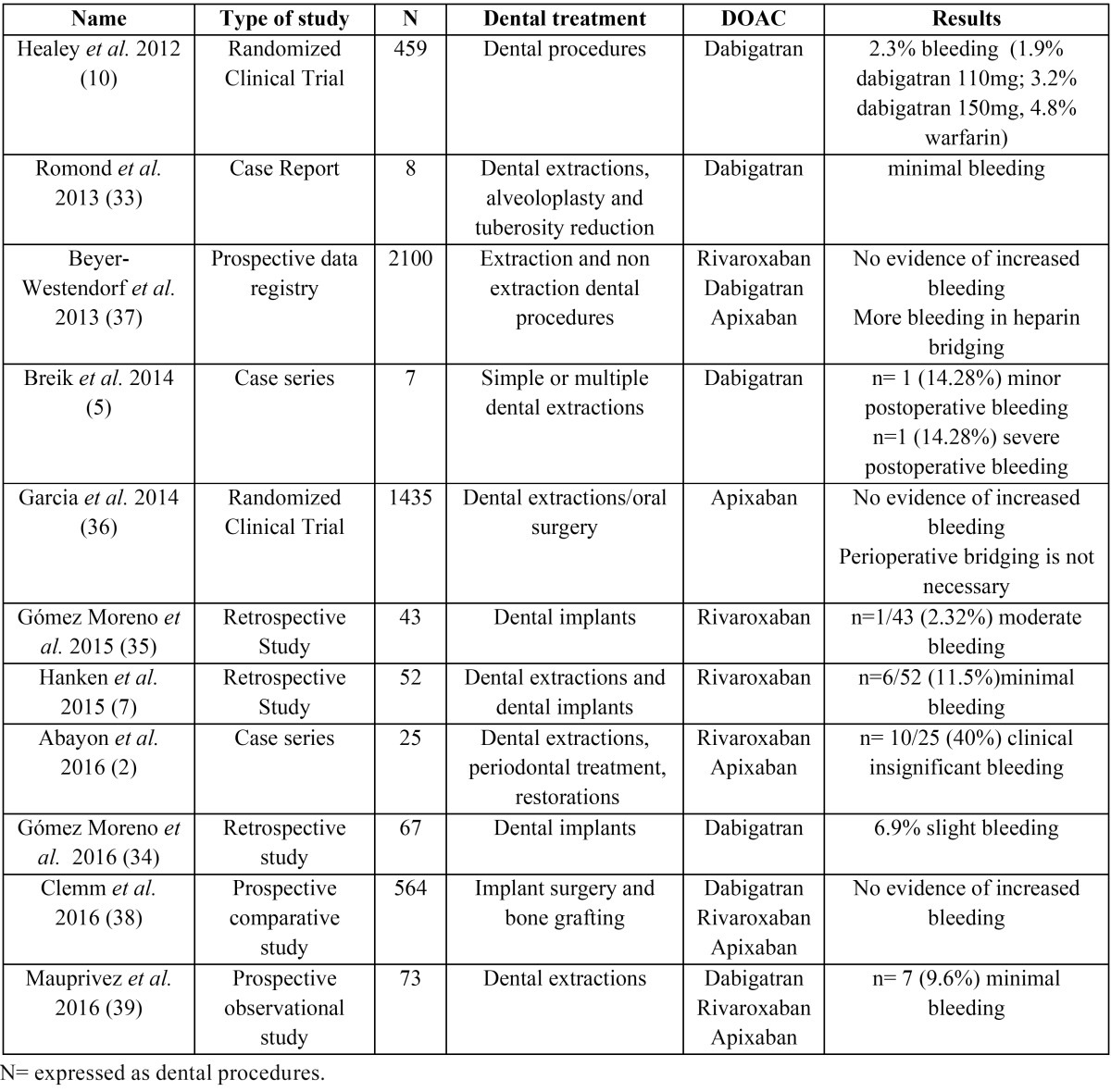
Studies included in the literature review.

**Table 3:**
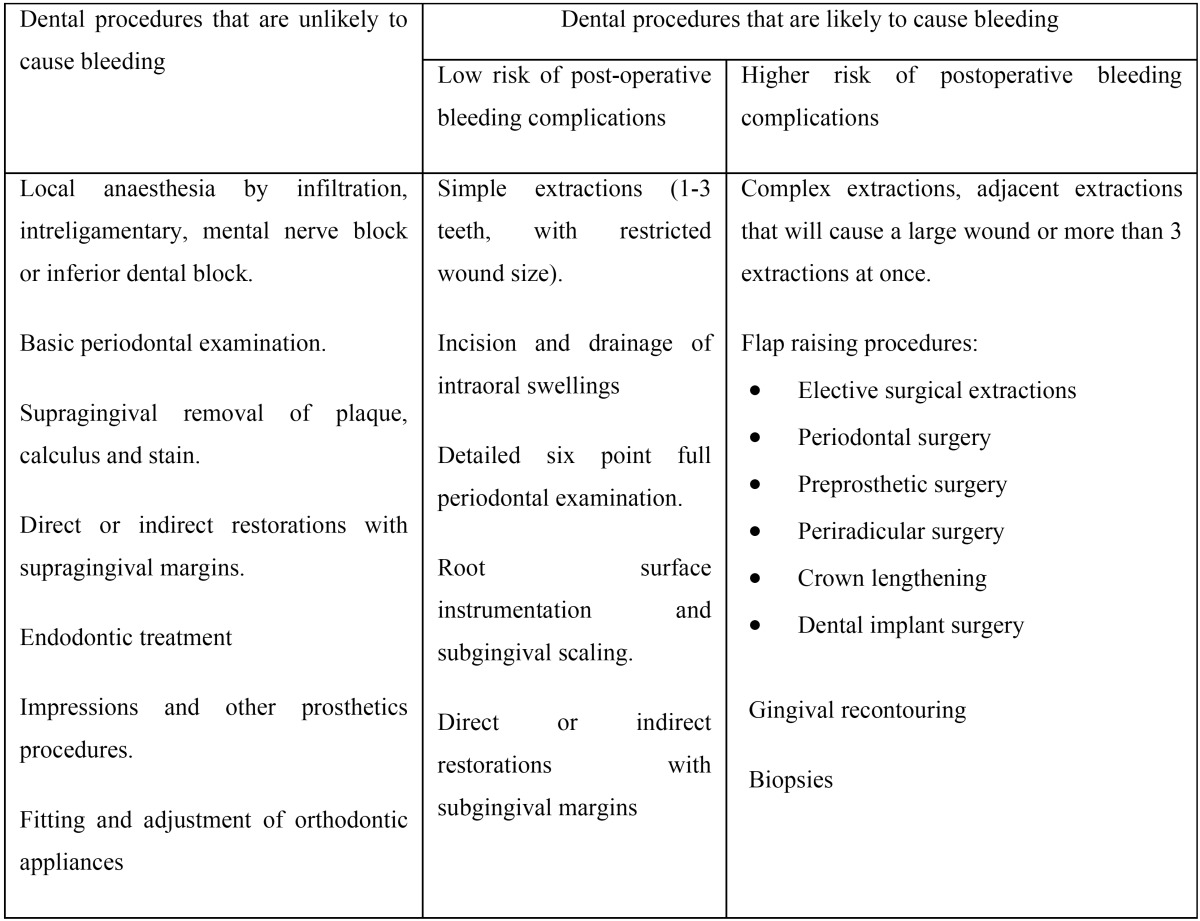
Post-operative bleeding risks for dental procedures (41).

**Table 4:**
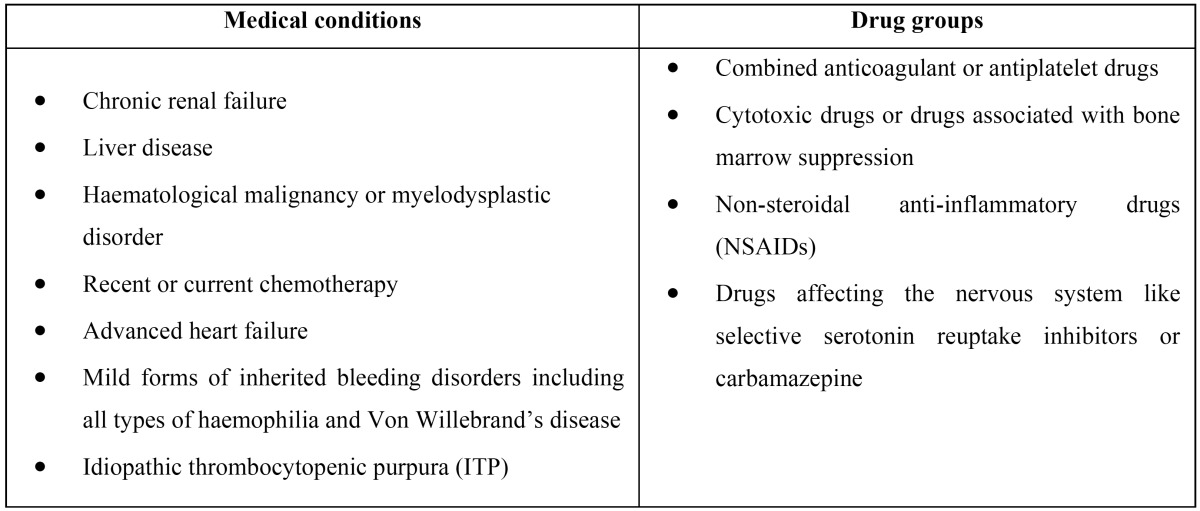
Medical conditions and drugs associated with increased bleeding risk (41).

## Discussion

There is no consensus in behalf of the protocol to follow in patients in treatment with DOACs who need a dental procedure or oral surgery. However, all the authors agree that it is necessary to individualize each case evaluating the difficulty of the procedure, the risk of bleeding, the risk of embolism and the renal function of the patient.

First of all, the articles that focus on the bleeding risk in patients treated with dabigatran have similar results and conclusions: oral procedures are safe and it is recommended to stop the medication between 12 and 48 hours before the procedure.

The first one, the randomized clinical trial published by Healey *et al.* (10) in 2012, compared the bleeding risk in patients treated with dabigatran and warfarin from the RE-LY trial. The 25% (n=4591) of patients in each group underwent surgery over a 2-year period and 10% of these underwent dental procedures. The drugs were discontinued prior to surgery, with the last dose of dabigatran given 35–85 h (mean 49 h) prior to the procedure, compared to 87–144 h (mean 114 h) for warfarin. There was no difference in peri-procedural bleeding between the two groups. The authors concluded that are similar rates of post-surgery bleeding events and thrombotic complications in patients in both groups. The authors recommended that for standard bleeding risk procedures, dabigatran should be stopped 2–3 half-lives before the procedure, and for high bleeding risk procedures, dabigatran should be stopped 4–5 half-lives prior to the procedure.

The case control published by Romond *et al.* (33) in 2013, goes in the same direction. The subject of study is a male patient of 67 year-old treated with 150mg dabigatran twice a day resumed 1 day after the surgery. After 8 surgical dental extractions the patient experienced minimal bleeding reported at the first week follow up. The author’s conclusions suggest that in dental extractions under dabigatran there is no greater risk of bleeding but there should be a consensus between the dentist and the physician responsible for the patient regarding the management of the anticoagulant treatment.

In 2015, Breik *et al.* (5) published a case series of 5 patients undergoing simple or multiple dental extractions treated with dabigatran 220mg/day. The proposed author’s protocol in general dental procedures like single dental extractions, periodontal treatment or root canal treatment is not to stop dabigatran. The authors also recommended after a dental extraction to take haemostatic measures such as mechanical pressure, suture and local haemostatic. In multiple extraction procedures, it is recommended a consensus with the physician responsible for the patient and stop dabigatran 24-48 hours before the procedure and recommence 24-48 hours post-procedure.

The last study of dabigatran was published in 2016 by Gomez Moreno *et al.* (34). This article is about dental implant placement in patients taking dabigatran 150mg twice a day. They found no statistically significant difference in the bleeding events between the dabigatran and the control group. They concluded that dental implant surgery can be safely performed in which the last dosage of dabigatran was taken at least 12 hours before the procedure, always applying local haemostatic measures (34).

Although the studies have very different characteristics, the dental procedures performed are varied and even the doses vary, dabigatran seems to be a safe drug that does not produce major haemorrhagic complications whether it is maintained or withdrawn the last dose. It may also be noted that in the case of complex procedures it would seem advisable to establish an anticoagulant treatment plan agreed with the patient’s reference physician

If we look at articles that evaluate the effects of rivaroxaban, there are some differences regarding the postoperative bleeding risk between the articles reviewed. While Gómez Moreno *et al.* (35) and Abayon *et al.* (2) found that there was no difference in pos-toperative bleeding, Hanken *et al.* (7) concluded that rivaroxaban has a higher risk of bleeding than other oral anticoagulants.

The retrospective study conducted in 2015 by Gómez Moreno *et al.* (35) evaluated dental implant surgeries in patients taking rivaroxaban without any modification or withdrawal of the drug. The postoperative measures performed were non absorbable suture and pressure with a gauze impregnated in 5% tranexamic acid during 30-60 minutes. There was no statistically significant difference between the rivaroxaban and the control group in the postoperative bleeding. They concluded that dental implant surgery in patients taking rivaroxaban is safe, with no need to interrupt or modify the medication and emphasize the importance to perform postoperative local hemostatic measures. Moreover, they highlight that dental implant placement has less risk than a dental extraction.

In the same line of conclusions is the case series of 9 patients published by Abayon *et al.* (2) in 2016. The subjects evaluated were in treatment with rivaroxaban 20mg hours that continued, partial interrupt or complete interrupt the anticoagulant treatment. They conducted all types of dental treatment (dental extractions, dental prophylaxis, dental restorations or periodontal treatment). They concluded that dental treatment may be safely performed following the three strategies, but they pointed out that larger scale comparative studies are necessary to assess the preferred approach.

On the other hand, we have the retrospective study published by Hanken *et al.* (7) in 2015. This study evaluated 337 oral procedures (327 dental extractions and 10 dental implants) in patients in treatment with Rivaroxaban 20mg/day without stopping the medication comparing with healthy subjects. They concluded that Rivaroxaban increases postoperative bleeding risk to 11.5%, which is a higher level compared to other oral anticoagulants. However, all the bleeding events were manageable with local compression, fibrin glue and secondary suture.

In the case of rivaroxaban the conclusions regarding bleeding rate are not homogeneous since there is at least one study in which it has been evidenced that the patients have a greater tendency to haemorrhage although the management is affordable.

There are two studies that evaluate the bleeding risk of apixaban. First, Garcia *et al.* (36) compared the bleeding risk in patients treated with apixaban and warfarin from the ARISTOTELE trial. Garcia *et al.* (36) used data from this trial to identify all patients who ‘‘underwent a procedure for which anticoagulant therapy would, in some clinical situations, be interrupted’’. Dental extraction/oral surgery accounted for 14.6% of all procedures. The authors concluded that the risk of or major bleeding in the procedures was low and similar rates of major bleeding events and thromboembolism took place in both groups during the 30 days after procedures. Abayon *et al.* (2) performed a study which includes a group of patients with different types of dental interventions as we have noted above, in which three different strategies are established: regarding anticoagulation continued, partial interrupt or complete interrupt, and there are not differences in bleeding.

Despite the limited data available, we can extract that apixaban is at least as safe as warfarin in terms of bleeding and the appearance of new cardiovascular events; and that remove or maintain the drug in dental procedures does not imply a higher rate of haemorrhagic complications.

Finally, of the reviewed, there are other articles that compare different types of oral anticoagulants, like warfarin and all kind of DOACs and also take into account anticoagulant withdrawal bridged with heparin.

In 2013, Beyer-Westendorf *et al.* (37) analysed data from a prospective registry of over 2100 patients to study the peri-procedural management of DOACs. The patients in the study were taking rivaroxaban (76%), dabigatran (23.5%) and apixaban (0.5%). Procedures were categorized into minimal procedures (including nonextraction dental procedures) and minor procedures (including dental extractions). The authors analysed the role of heparin bridging in this type of patients and concluded that the prevalence of bleeding was found to be higher in the groups of patients who had received heparin bridging, as might be expected, without affecting the risk of a thromboembolic event. They concluded that heparin bridging was therefore not recommended in the peri-procedural management of patients taking DOACs, and short-term interruption appeared to be safe in the minimal and minor surgery groups. In the same line Garcia *et al.* (36) concluded that heparin bridging was not recommended in the peri-procedural management of patients taking apixaban and Clemm *et al.* (38) that the heparin bridging may be associated with a higher postoperative bleeding risk.

In 2016, Clemm *et al.* (38) conducted a prospective comparative study to analyse the postoperative bleeding risk in of patients undergoing dental implant and bone graft surgeries without stopping their anticoagulation treatment. They compared patients treated with i) platelet aggregation inhibitors, ii) vitamin K inhibitors, iii) vitamin K inhibitor withdrawal bridged with heparin and iv) direct oral anticoagulants. The percentage of patients that experienced postoperative bleeding was 1.2%. None of the patients with DOACs presented bleeding, and the group with more frequency of bleeding was the vitamin K withdrawal bridged with heparin. They concluded that the bleeding risk after implant surgery and bone grafting procedures is very low in patients conti-nuing the anticoagulation treatment, that DOACs do not significantly increase this risk.

Also in 2016 Mauprivez *et al.* (39) conducted a prospective observational study to compare the incidence of postoperative bleeding after dental extractions between patients treated with DOACs and patients treated with Vitamin K antagonists without withdrawal of the anticoagulant treatment. The bleeding events that took place were mild and easily controlled and were comparable between the two groups. They concluded that dental extractions can be safely performed in patients treated with DOACs without interrupting or modifying the dose.

Although the patient groups and their medications are very heterogeneous, these findings are in agreement with the previously mentioned study and we can conclude that the new anticoagulants compared with the vitamin K antagonists do not seem to produce greater haemorrhagic complications.

We can also extract from various studies (Garcia *et al.* (36) Beyer-Westendorf *et al.* (37) and Clemm *et al.* (38) that heparin bridging was not recommended in the peri-procedural management of patients taking DOACs because may be associated with a higher postoperative bleeding risk.

The latest quality non-systematic reviews published by Elad (12) and Johnston (40) in 2016 have similar conclusions to our review and agree that there is lack of scientific evidence in order to establish a protocol for dental procedures in patients treated with DOACs and that more comparative clinical trials are needed. In both reviews is pointed out that, before the dental procedure, it is important to assess the type of procedure, the extent of expected bleeding and the medical background of the patient. Moreover, whether to discontinue or not the anticoagulant treatment, there is consensus that the preferred approach is to continue the drug or time the dental treatment as late as possible after the las DOACs dose; since local haemostatic measures are likely to be sufficient and the risk of a thromboembolic event if DOACs are stopped is higher than the bleeding risk after a dental procedure.

All these recommendations are in the same line with the guideline of the NHS and the Scottish Dental Clinical Effectiveness Program on the Management of Dental Patients Taking Anticoagulants or Antiplatelet Drugs, summarized by Daly (41). This paper suggests two different approaches depending on the type of dental procedure and the risk of bleeding based on certain clinical characteristics of patients which are summarized in tables 3 and 4. In dental procedures with low risk of bleeding complications it is not necessary to interrupt the anticoagulant medications. However, in dental procedures with higher risk of bleeding complications, it is recommended to advise the patient to miss or delay their morning dose on the day of the dental treatment. The patients can restart their medication only when haemostasis has been achieved.

Based on the studies analysed we can support the following conclusions regarding the use of DOACs in dental practice,

DOACs are relatively safe drugs in terms of bleeding. They can be compared with classic oral anticoagulants. Removal of the anticoagulant a few hours before the procedure appears to be an adequate approach that does not increase the risk of thromboembolic events. However when dental procedures are performed without withdrawal of the drug the bleeding is manageable with conventional haemostasis measurements. The bridging approach with heparin does not seem to be recommended in any case because precisely these changes in anticoagulant treatment appear to increase post-operative bleeding.

In complex patients, with clinical characteristics predisposing to bleeding and even in complex and invasive dental procedures it is necessary an individualized approach with the consensus between the dentist and the patient´s responsible physician in order to minimize risks and possible complications.

The available scientific evidence regarding the management of these drugs in the dental office is very weak and therefore will be necessary to carry out solid clinical trials to confirm these initial conclusions.
